# Seed Priming Modulates Physiological and Agronomic Attributes of Maize (*Zea mays* L.) under Induced Polyethylene Glycol Osmotic Stress

**DOI:** 10.1021/acsomega.3c01715

**Published:** 2023-06-15

**Authors:** Hussain Ahmad Kakar, Sami Ullah, Wadood Shah, Baber Ali, Sanam Zarif Satti, Rehman Ullah, Zahir Muhammad, Sayed M. Eldin, Iftikhar Ali, Mona S. Alwahibi, Mohamed S. Elshikh, Sezai Ercisli

**Affiliations:** † Department of Botany, University of Peshawar, Peshawar 25120, Pakistan; ‡ Biological Sciences Research Division, Pakistan Forest Institute, Peshawar 25120, Pakistan; § Department of Plant Sciences, Quaid-i-Azam University, Islamabad 45320, Pakistan; ∥ Future University in Egypt, Center of Research, Faculty of Engineering, New Cairo 11835, Egypt; ⊥ University of Swat, Centre for Plant Science and Biodiversity, Charbagh 19120, Pakistan; # Department of Genetics and Development, Columbia University Irving Medical Center, New York, New York 10032, United States; ∇ Department of Botany and Microbiology, College of Science, King Saud University, Riyadh 11451, Saudi Arabia; ○ Department of Horticulture, Agricultural Faculty, Ataturk Universitesi, Erzurum 25240, Türkiye; ◆ HGF Agro, Ata Teknokent, TR-25240 Erzurum, Türkiye

## Abstract

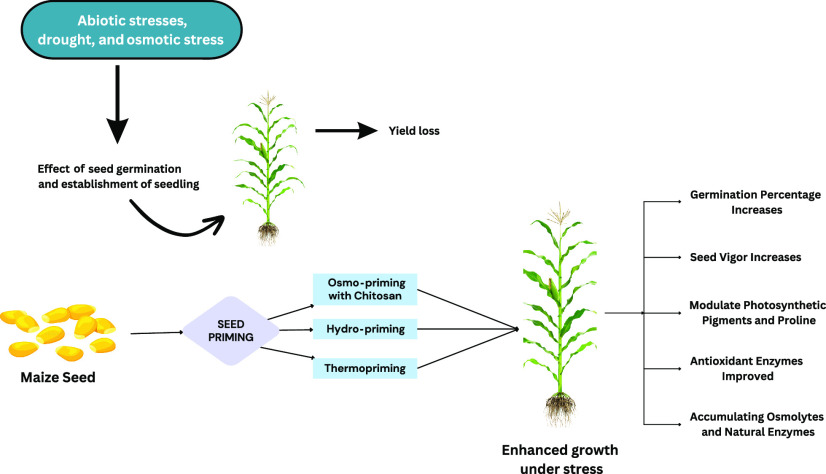

Drought and osmotic stresses are major threats to agricultural crops as they affect plants during their life cycle. The seeds are more susceptible to these stresses during germination and establishment of seedlings. To cope with these abiotic stresses, various seed priming techniques have broadly been used. The present study aimed to assess seed priming techniques under osmotic stress. Osmo-priming with chitosan (1 and 2%), hydro-priming with distilled water, and thermo-priming at 4 °C were used on the physiology and agronomy of *Zea mays* L. under polyethylene glycol (PEG-4000)-induced osmotic stress (−0.2 and −0.4 MPa). The vegetative response, osmolyte content, and antioxidant enzymes of two varieties (Pearl and Sargodha 2002 White) were studied under induced osmotic stress. The results showed that seed germination and seedling growth were inhibited under osmotic stress and germination percentage, and the seed vigor index was enhanced in both varieties of *Z. mays* L. with chitosan osmo-priming. Osmo-priming with chitosan and hydro-priming with distilled water modulated the level of photosynthetic pigments and proline, which were reduced under induced osmotic stress; moreover, the activities of antioxidant enzymes were improved significantly. In conclusion, osmotic stress adversely affects the growth and physiological attributes; on the contrary, seed priming ameliorated the stress tolerance resistance of *Z. mays* L. cultivars to PEG-induced osmotic stress by activating the natural antioxidation enzymatic system and accumulating osmolytes.

## 1 Introduction

Drastic shifts in environmental regimes are becoming a hindrance in achieving the growing demand of food and acquiring sustainable agriculture.^1,2^ Climatic changes lead to fluctuations in temperature,^3^ droughts, floods, and other environmental calamities, eventually leading to decrease crop productivity.^4^ The abiotic stresses such as drought,^5^ extreme temperature, frost, heavy metals,^6−15^ and salinity^16,17^ severely impair plant growth and productivity worldwide.^18,19^ Drought, being the most important environmental stress, severely damages plant growth and development.^20^ Salt stress leads to an imbalance between antioxidant concentrations and reactive oxygen species (ROS) levels, thus resulting in oxidative stress.^21^ Salinity-induced production of reactive oxygen species (ROS) causes damage to mitochondria and chloroplasts.^22,23^ Salt stress adversely affects almost every aspect of the physiology and biochemistry of plants and significantly reduces yield, the most serious threat to agriculture and major environmental factor that limits crop growth and productivity.^24,25^ Drought and salinity stresses lead to another abiotic stress, the “osmotic stress”. Osmotic stress severely affects plants during their life cycle; it results in leaf chlorosis and antioxidant’s imbalance.^24^ However, reduction in growth depends upon the duration and the severity of stress.^26−29^ In this connection, many scientific studies have concluded that osmotic stress hampers the growth of leaves, stems, roots, and total plant dry mass.^25,30^

Drought stress is a major threat for agricultural crops as the population of the world is increasing at an alarming rate, thus fulfilling their water demand that leads to worsening of the water deficit condition.^31^ Drought stress is the major cause of crop loss as it reduces yield components, such as reduction in the leaf size and number of grains.^32^ A major proportion of agriculture land is affected with varying degrees of drought and low atmospheric humidity leading to drought, which is the limiting factor for better plant performance and higher crop yield.^33,34^ A proper amount of soil moisture is compulsory for plant growth, transpiration, and also for transportation of food prepared in the process of photosynthesis in leaves.^35^ A number of strategies have been devised to overcome the adverse effects of abiotic stresses, such as the selection *in vitro* propagation of drought resistant cultivars,^36^ germplasm,^37^ and plant breeding methods,^38^ but all these strategies are expensive. However, an alternative strategy for the possibilities to overcome salt and drought stresses is seed priming. Nowadays, seed priming techniques such as hydro-priming, osmo-priming, thermo-priming, and hormonal priming have been used to enhance emergence of roots and shoots, attaining vigorous plants, and better drought tolerance in many field crops,^39^ such as wheat, maize, chickpea,^40^ sunflower,^41^ and cotton.^42^

Salinity is the buildup of soluble salts by which saline soils are formed, and the concentration of salt in soil is above the normal levels.^43^ Salinity is one of the most serious factors, which limits the growth and development of plants.^44,45^ It adversely affects seed germination, plant vigor, and crop yield.^25^ Salinity may be due to many factors, but some of the adverse effects of salinity have been attributed to increase in sodium and chloride ions in different plant organs; hence, these ions create the critical conditions for plant survival by interpreting different vital plant mechanisms.^46,47^ Sodium and chloride are the major ions, which cause many physiological disorders in citrus and limit plant growth and productivity.^48^ Excess of these salts also enhances the osmotic potential of the soil matrix as a result of which water intake by plants is restricted.^49^

Salinity stress reduces the chlorophyll content of sensitive species more in comparison to tolerant species.^50,51^ The most negative effect on seed germination is the presence of salts in the soil.^52^ It has been reported that not only do the differences in plant response to the amount of salt available in soil and irrigation water depend on plant species, but crop development stages and seedling growth stages are also the most vulnerable stages in the life cycle of plants.^53^ Therefore, these stages are focused and taken into consideration when the salt tolerance potential of a plant is determined.^54^ Over 6% of the world’s total land area and 20% of the irrigated land area are affected by salinity stress. Salinity has reached a level of 19.5% in all the irrigated lands (out of 230 million hectares of irrigated land, 45 million hectares are salt-affected soils) and 2.1% in dry lands worldwide. Almost 20% of the cultivated area of the world and half of the world’s irrigated lands are stressed with salinity.^55^

The condition in which water is deficit due to high levels of salinity or drought is known as osmotic stress, and it creates ion toxicity and disturbs ionic balance.^56^ Osmotic stresses affect plants during their life cycle as seeds are mostly susceptible to these stresses between sowing and seedling establishment.^57^ Germination and seedling growth of the plants decrease due to nutritional imbalance, similarly in saline conditions due to an external osmotic potential that prevents water uptake or due to the toxic effects of Na^+^ and Cl^–^ ions or both on the germination of seeds.^58^ Salinity and drought stress reduce the plant growth and development through specific ion effects, nutritional imbalance, low osmotic potential of soil solutions, and combination of all these factors.^59^ Osmotic stress can affect various major plant processes like photosynthesis, protein synthesis, and lipid metabolisms. Generally, salt stress causes both osmotic stress and ionic stress.^60^ The osmotic effect initially reduces the ability of the plant to absorb water.^61^ Several minutes after the initial decrease in leaf growth, a gradual growth recovery takes place until a new steady state is reached, depending on the salt concentration outside the root.^25^

Osmotic stress disturbs plants’ physiological and biochemical processes due to water stress conditions, which is related to a decrease in rate of photosynthesis, closing of stomata, ultimately interrupting photosynthetic pigments and protein formation.^62^ A reduction in net photosynthetic rate under drought stress conditions is also related to disturbances in biochemical processes of a non-stomatal nature, caused by oxidation of chloroplast, lipids, and changes in the formation of pigments and proteins.^63,64^

In seed priming, seeds are pre-soaked in distilled water or osmotic solutions. Seed priming is a simple, cost-effective, and compelling approach employed for the enhancement of swift seed germination, early seedling growth, and improved yield under normal and stressed conditions.^65^ Priming is a form of seed preparation in which seeds are pre-soaked before planting.^66^ To enhance the resistance of plants to abiotic stresses, the seed priming technique is being used. In this technique, the seeds are soaked in various solutions or exposed to varying degrees of temperatures prior to sowing. Seed priming with organic and inorganic compounds, antioxidants, and hormones have insured an extensive survivability in crop plants under osmotic stress.^65^ Seed priming is an affordable, economical, and effective scientific procedure for the improvement of seed germination, early seedling, and yield under osmotic stress conditions.^67^

The chitosan α,β-(1,4)-glucosamine polymer is a safe, natural, and cheap polysaccharide and is produced from chitin, which is the major structural component of the fungus cell wall and the exoskeleton of arthropods.^68,69^ Chitosan can be used as plant fertilizer as it promotes seed germination, enhances germination percentage, and can modulate the responses of plants to abiotic stresses.^70,71^ Chitosan priming improves maize germination and seedling growth in relation to physiological changes under low temperature stress; it is used as osmo-priming to decrease the adverse effect of abiotic stress. Chitosan is obtained by deacetylation of chitin.^72^

The present research work was aimed to study the effect of various priming techniques on *Zea mays* L. under induced PEG osmotic stress. The effects of chitosan as osmo-priming, distilled water as hydro-priming, and thermo-priming on the physiological and agronomic performance of two cultivars of *Z. mays* L. were assessed, and a comparison was done between these priming techniques under polyethylene glycol (PEG-4000)-induced osmotic stress. The efficacy of priming techniques in regulating the key metabolic activities improves the osmotic stress tolerance capacity of maize cultivars subjected to varying levels of induced (PEG) osmotic stress conditions.

## 2 Materials and Methods

### 2.1 Soil Elemental Analysis

The soil texture class was calculated as silt loam. Soil pH was 6.0, electrical conductivity was 2.41 ds/m,^73^ soil nitrogen (N) content was 2.05 g/kg, organic carbon (C) content was 20.5 g/kg, and potassium (K) available was 90.5 mg/kg.^74^

### 2.2 Area of Study and Experimental Design

**Figure 1:**
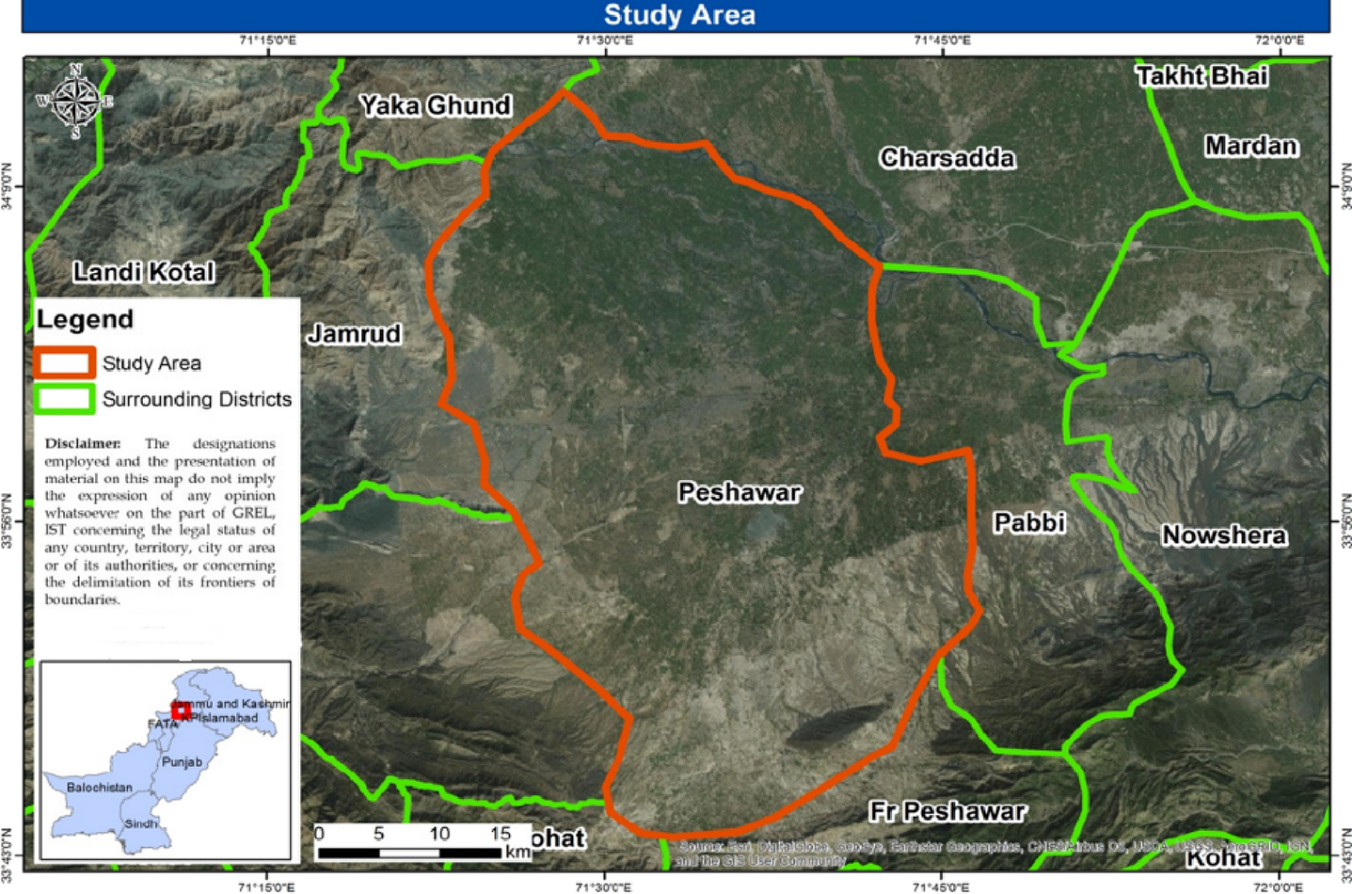
Spatial map of the study area.^75^

**Figure 2:**
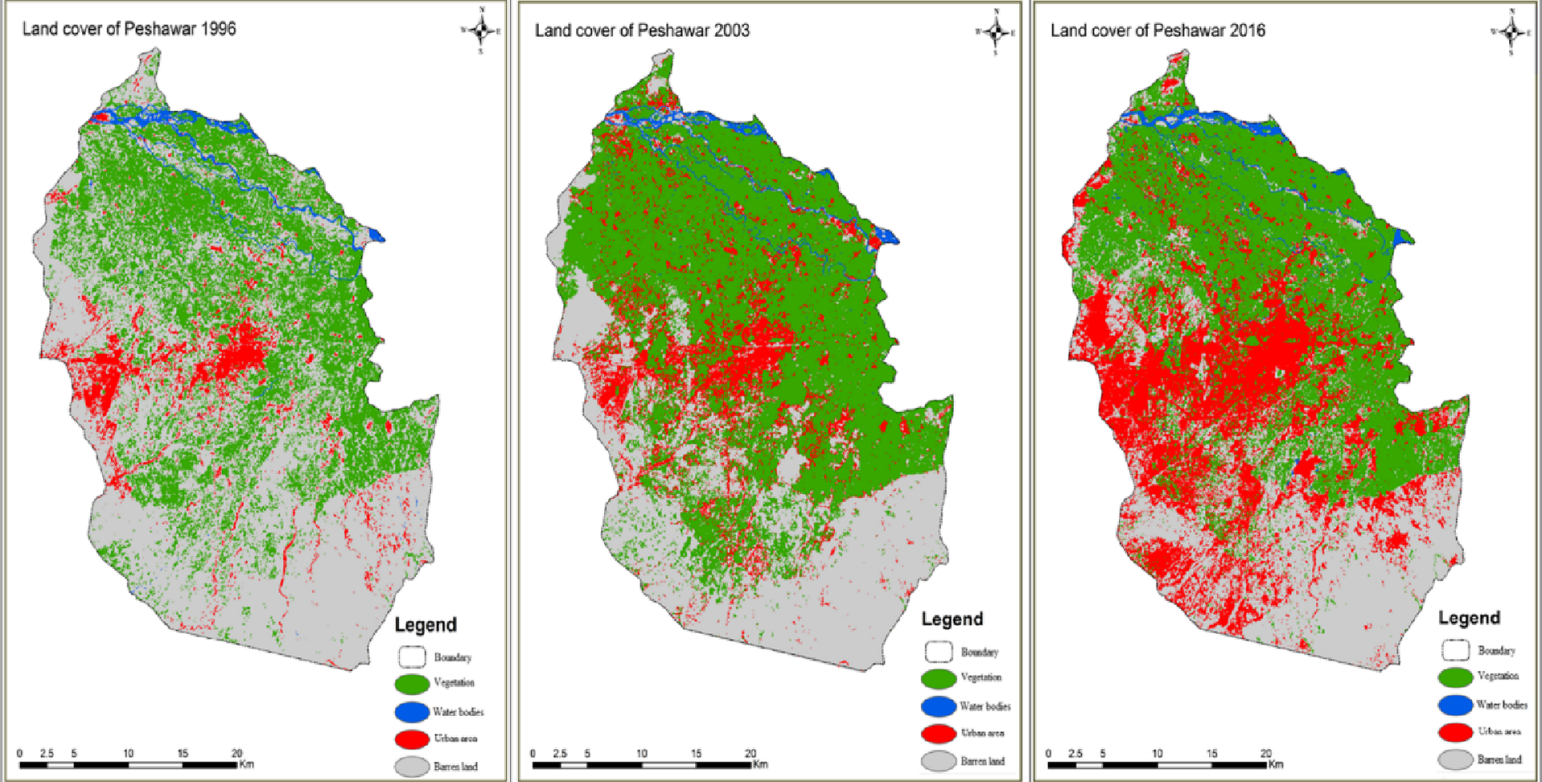
Land cover of Peshawar since 1996, 2003, and 2016.^75^

The pot experiment was conducted at the green house of the Department of Botany, University of Peshawar, Pakistan during the month of February 2018. Peshawar is located in the Iranian plateau having tropical climatic conditions. It is the largest and capital city of the Khyber Pakhtunkhwa province of Pakistan (Figure 1). The temperature of Peshawar ranges from 5 °C (in January) to 39 °C (in June). The total area of Peshawar is 1257 km^2^ with an elevation of 340 m/L, 115.49 feet. The images in Figure 2 depict four land use classes including vegetation, water bodies, urban area, and barren land during the years 1996, 2003, and 2016.

Seeds of two cultivars (Pearl and Sargodha) of maize (*Z. mays* L.) were collected from the National Agriculture Research Centre, Islamabad (NARC). The temperatures of Peshawar in the month of February were 20 and 7 °C (high/low), humidity was 61%, and light duration was 10.9 h/day. Before sowing, the seeds were surface-sterilized with 70% ethanol and 0.1% mercuric chloride solution. After surface sterilization, the seeds were rinsed with distilled water. In addition, identical sized and smooth surfaces seeds were selected for the proposed trial. Ten pre germinated seeds of each cultivar (Pearl and Sargodha) both primed and non-primed were sown in pots. Watering of pots was done during the whole growing season regularly. For better growth of seedlings, the pots were exposed to sunlight and kept free from weeds by uprooting weeds periodically. Out of the total 90 pots, 30 pots were taken as the control group and the remaining 60 pots were applied with different levels of osmotic stresses (−0.2 and −0.4 MPa) using polyethylene glycol (PEG) 4000. Three replicates of each treatment were exposed to osmotic stress. During the entire experiment, all the standard practices were done from time to time. The plants were then harvested and frozen for the evaluation of physiological and agronomic studies in the laboratory.

### 2.3 Osmo-Priming

For osmo-priming, the seeds were primed with chitosan (1 and 2% solution) for 3 h followed by washing with distilled water thrice and kept for a period of 2 days in the oven for the purpose of drying at 26 ± 2 °C.^76^

### 2.4 Thermo-Priming

Thermo-priming of seeds was done by keeping the seeds at 4 °C for 1 h in the freezer. The seeds were then washed with distilled water and kept in the oven for a period of 2 days for the purpose of drying at 26 ± 2 °C.^77^

### 2.5 Hydro-Priming

For hydro-priming, the seeds were put in distilled water and soaked for 24 h. The seeds were then filtered and kept for 2 days in the microwave oven at a temperature of 26 °C for drying.^77^

### 2.6 Induction of Osmotic Stress

Osmotic stress was induced using polyethylene glycol (PEG) 4000. PEG solution (20 mL) was directly given to pots after sowing of seeds. PEG 4000 solution was prepared using the standard procedure in ref (78). PEG (14 g) was dissolved in 100 mL of distilled water to induce −0.2 MPa pressure. PEG 4000 (28 g) was dissolved in 100 mL of distilled water to induce −0.4 MPa pressures.^79^

### 2.7 Agronomic Characteristics

#### 2.7.1 Absolute Growth Rate (AGR)

Absolute growth rate is the total growth rate per unit of time. After 10 days of induction of osmotic stress, three replicates of each treatment were taken to find the mean of each measurement with the help of the following formulas. Absolute growth rate (AGR) was calculated with the help of the formula recommended in ref (80).
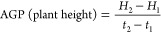

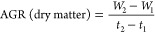
where *H*_1_ and *H*_2_ are the plant heights (cm). *W*_1_ and *W*_2_ are the plant dry weights at time *t*_1_ and *t*_2_, respectively.

#### 2.7.2 Relative Growth Rate (RGR)

Relative growth rate is the rate of growth with respect to its initial size. Relative growth rate (RGR) of plant growth was calculated with the help of the formula as described in ref (80).
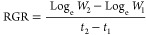
where *W*_1_ and *W*_2_ are the plant dry weights (g) during time *t*_1_ and *t*_2_, respectively. Log_e_ is the natural logarithm (logarithm to the base of 2.3026). Plant relative growth rate is shown in g/plant/day.

#### 2.7.3 Net Assimilation Rate (NAR)

NAR is the rate of an increase in dry weight per unit of leaf area. NAR was calculated using the equation arranged in ref (80). It refers to any increase in the dry matter of plant per unit of its assimilatory surface area of per unit of time.
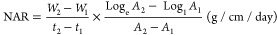
where *A*_1_ and *A*_2_ are the surface areas of leaves and *W*_1_ and *W*_2_ are the total plant dry matters at *t*_1_ and *t*_2_ time, respectively.

#### 2.7.4 Crop Growth Rate (CGR)

Crop growth rate is the gain of dry matter production of the crop in a given land per unit of time. The crop growth rate was determined using the formula described in ref (81). The samples were kept in the oven up to 3 days at 30 °C, and the dry weight was calculated.
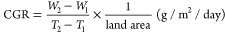
where *W*_1_ and *W*_2_ are the plant dry weights during time *T*_1_and *T*_2_, respectively.

#### 2.7.5 Leaf Area Ratio (LAR)

The leaf area ratio is the ratio between the areas of leaf lamina to the total biomass of plants. The leaf area ratio (LAR) was determined by the method established in ref (81). Length and width of leaves were measured with the help of a measuring scale. The sample in the oven at 30 °C for 48 h and plant dry mass (g) were determined.
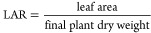


#### 2.7.6 Root Shoot Ratio (RSR)

The root shoot ratio is the ratio of root weight to the plant top weight. The root shoot ratio (RSR) was reported as per the formula suggested in ref (81). The samples were kept in the oven for 48 h, and dry masses (g) of both root and shoot were determined.
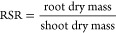


#### 2.7.7 Percent Moisture Content (PMC)

The percent moisture content is the percentage of the amount of moisture present in plant parts like shoot, leaf, root, and soil. The moisture content percentage of shoot, leaf, root, and soil was calculated as the formula suggested in ref (81).
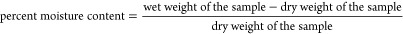


#### 2.7.8 Final Emergence Percentage (FEP)

Final emergence percentage is the percentage of seeds germinated of the total number of seeds initially sowed. FEP was calculated using the formula as given in ref (81). It is the percentage of total seeds emerged.
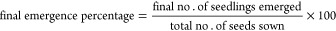


#### 2.7.9 Seed Vigor Index (SVI)

The seed vigor index is the total activities of a seed during germination and seedlings. The seed vigor index was determined using the method in ref (82).
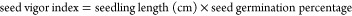


#### 2.7.10 Coefficient of Velocity of Germination (CVG)

The coefficient of velocity of germination indicates the rapidity of germination of seeds. CVG represent germination velocity. If the time that is required for germination is low, then the value of the CVG will be greater. When all the seeds germinate, we will have highest value of CVG.^82^
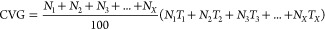
where *N* is the number of seeds that is germinated per day and *T* represents the time period, which is considered in days from seed sowing.

#### 2.7.11 Leaf Area Index (LAI)

The leaf area index is the quantification of the leaf area under canopy. The leaf area was determined using the formula proposed in ref (83).
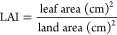


#### 2.7.12 Mean Emergence Time (MET)

Mean emergence time is the total time taken by a seed to germinate. Mean emergence time shows how many inhabitants have emerged faster. The greater the germinated inhabitants, the lower the mean germination time.^83^
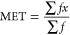
where *f* shows the numbers of seeds germinated on *x* day.

#### 2.7.13 Timsen Germination Index (TGI)

The Timsen germination index is the germination of seeds per day or in a given time. The TGI indicates the number of seeds germinated on each day. It was determined using the equation used in ref (83).
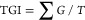
where *G* is the principal germination percentage per day whereas *T* is the entire germination period.

### 2.8 Physiological and Biochemical Attributes

#### 2.8.1 Total Chlorophyll Content Determination (TCC)

The photosynthetic pigments were quantified by following refs (84) and (85). These pigments were extracted by homogenizing 0.1 g of fresh leaves with 6 mL of 80% ethanol. The extract was centrifuged, and the supernatant was taken in test tubes. A spectrophotometer (752 N UV–vis, Beijing, China) was used to evaluate the optical density of chlorophyll a and b and carotenoids at 663, 645, 510, and 480 nm.
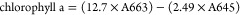

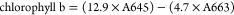

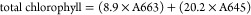

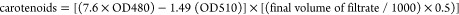


#### 2.8.2 Estimation of the Soluble Protein Content (SPC)

Bovine serum albumin (BSA), as described by Mendez and Kwon,^86^ was used as a reference to assess the protein content. The fresh leaves (0.1 g) were crushed in a mortar and pestle with 1 mL of phosphate buffer (pH 7.5) and centrifuged for 10 min at 3000 rpm. The total volume of the supernatant (0.1 mL) in test tubes was increased to 1 mL by adding distilled water. Reagent C (solution a and b in a 50:1 ratio) (solution a: 2% Na_2_CO_3_, 1% Na-K, 0.4% 0.1 N NaOH; solution b: 0.5% CuSO_4_·5H_2_O in dH_2_O) (1 mL) was added and mixed for 10 min, and then, 0.1 mL of reagent D (Folin phenol: distilled water in a 1:1 ratio) was added. The different concentrations (20, 40, 60, 80, 320, and 640 mg) of the BSA solution were prepared, and then, the absorbance of all samples was measured at 650 nm after 30 min of incubation.

#### 2.8.3 Quantification of the Total Proline Content (TPC)

The proline content in shoots was measured using the technique proposed by Parveen and Siddiqui.^87^ Fresh shoot material (0.2 g) was crushed in 3 mL of 3% sulfosalicylic acid and stored at 5 °C overnight. The obtained suspension was centrifuged for 5 min at 3000 rpm. The supernatant (2 mL) was blended with an acidic ninhydrin reagent after centrifugation. This reagent was prepared by dissolving 1.25 g of ninhydrin in 20 mL of phosphoric acid (6 M) and 30 mL of glacial acetic acid (1 M H_3_PO_4_ = 3 N H_3_PO_4_) with constant stirring. The reagent was kept stable for 24 h. The tubes carrying the contents were heated for 1 h in a water bath at 100 °C. After cooling, the mixture was extracted with 4 mL of toluene in a separate funnel. At 520 nm, optical density was determined using toluene as blank.
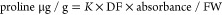
where *K* = 17.52, dilution factor = 2, and fresh weight = 0.5 g.

#### 2.8.4 Quantification of the Total Soluble Sugar Content (TSC)

Total soluble sugars (TSS) were calculated using the Grad’s method.^88^ The fresh leaves (0.1 g) were homogenized with 3–5 mL of 80% ethanol to eliminate all traces of soluble sugars and centrifuged for 10 min at 10,000 rpm. The supernatant was collected and processed to calculate TSS. A freshly prepared anthrone solution (3 mL) and 0.1 mL of alcoholic extract were mixed in test tubes. All test tubes were heated for 12 min in boiling water and then iced for 10 min before being incubated for 20 min at 25 °C. The optical density of the solution was measured at 625 nm using a spectrophotometer (752 N UV–vis, Beijing, China). The total soluble sugars were estimated in μg/mL of fresh weight using the glucose standard curve. To generate a glucose standard curve, a stock solution of glucose was prepared in various concentrations (0, 20, 40, 60, and 100 mg), and optical density was measured at 625 nm. After absorbance, the regression model was used to generate a glucose standard curve.

#### 2.8.5 Quantification of Antioxidants

The antioxidants (POD, SOD, and APX) were evaluated in accordance with ref (89). The fresh leaf samples (0.2 g) were crushed in a 2 mL extraction buffer (potassium phosphate, pH 7.5) and ascorbic acid (1 mM) to determine the APX level. The crushed materials were centrifuged for 20 min at 4 °C and 13,000 rpm. The OD was obtained at 290 nm to evaluate APX. A standard curve was used to measure the activity in units/mg of proteins by estimating the decrement of ascorbate.

To estimate SOD, the plant leaves were crushed in 4 mL of solution (1 g of PVP, 0.0278 g of Na_2_EDTA) and centrifuged at 10,000 rpm. A reaction mixture (400 μL of H_2_O + 350 μL of phosphate buffer + 100 μL of methionine + 50 μL of NBT + 50 μL of enzyme extract + 50 μL of riboflavin) was prepared to measure the activity of the SOD enzyme. The mixture was then exposed to light for 15 min, with the decrease in absorbance measured at 560 nm. A blank was made by omitting the enzyme extract. The activities of SOD were then calculated and expressed in milligrams per milligram of the total soluble protein.

Using a precooled mortar and pestle, freshly procured plant leaves (0.20 g) were crushed in 3 mL of 100 mM phosphate buffer (PB) for the POD assay. To separate the homogenate, the sample extract was centrifuged at 4 °C and 10,000 rpm for 15 min. To determine peroxidase, an OD at 470 nm was obtained. One unit of POD is defined as the amount of enzyme that increases by 0.100 absorbance at 436 nm/min.

The methodology of ref (90) was followed for the estimation of CAT. Leaves (0.5 g) were homogenized in 1.0 mL of phosphate buffer, which is followed by addition of H_2_O_2_ (1 mL) and phosphate buffer (3.0 mL). The absorbency value was then recorded at 240 nm.

#### 2.8.6 Statistical Analysis

Statistical analysis was done by using IBM SPSSS Statistics 26, Excel, and ORIGIN 2021 PC Corporation. ANOVA with least significant difference (LSD) and principle component analysis (PCA) was applied to analyze the data. The post hoc test was used for significant difference and expressed in the form alphabetical letters on bars of figures.

## 3 Results

### 3.1 Agronomic Characters

The results of agronomic characteristics presented in Tables 1–4 included mean germination time (MGT), germination index (GI), absolute growth rate (AGR), relative growth rate (RGR), relative water content (RWC), leaf area ratio (LAR), root shoot ratio (RSR), moisture content percentage (MCP), and seed vigor index (SVI), and all these attributes showed high values with chitosan priming (1 and 2%) under induced polyethylene glycol (PEG) osmotic stress in both of the studied cultivars of *Z. mays* L. In pearl variety, osmo-priming proved to be effective, while in Sargodha 2002 White, variety treatment with hydro-priming showed significant (*p* < 0.05) results for the above-mentioned agronomic attributes.

**Table 1:** Effect of Osmo-Priming, Thermo-Priming, and Hydro-Priming on Absolute Growth Rate, Relative Growth Rate, Net Assimilation Rate, and Leaf Area Index under Polyethylene Glycol-Induced Osmotic Stress^a^

variety	treatment	treatment	AGR1	AGR2	RGR	NAR
Pearl	osmotic stress	control	0.13 ± 0.01	0.31 ± 0.06	0.98 ± 0.04	1.85 ± 0.04
		0.2 PEG	0.17 ± 0.01	0.43 ± 0.11	1.68 ± 0.10	4.90 ± 0.01
		0.4 PEG	0.18 ± 0.02	0.34 ± 0.07	2.32 ± 0.85	6.04 ± 2.55
	osmo-priming	1% chitosan	0.23 ± 0.01	0.67 ± 0.10	5.02 ± 0.93	15.2 ± 3.94
		1% chitosan + 0.2 PEG	0.19 ± 0.02	0.54 ± 0.09	5.42 ± 0.15	16.9 ± 1.26
		1% chitosan + 0.4 PEG	0.14 ± 0.02	0.31 ± 0.03	1.69 ± 0.48	2.92 ± 0.96
	osmo-priming	2% chitosan	0.20 ± 0.01	0.21 ± 0.04	1.15 ± 0.27	1.97 ± 0.68
		2% chitosan + 0.2 PEG	0.12 ± 0.03	0.25 ± 0.03	2.02 ± 0.64	4.39 ± 1.64
		2% chitosan + 0.4 PEG	0.18 ± 0.02	0.36 ± 0.14	3.18 ± 1.21	8.87 ± 4.29
	thermo-priming	4 °C	0.14 ± 0.01	0.24 ± 0.03	1.55 ± 0.30	3.13 ± 0.99
		4 °C + 0.2 PEG	0.14 ± 0.01	0.25 ± 0.03	1.46 ± 0.15	2.59 ± 0.24
		4 °C + 0.4 PEG	0.11 ± 0.01	0.27 ± 0.01	2.57 ± 0.44	5.61 ± 1.29
	hydro-priming	hydro	0.13 ± 0.01	0.22 ± 0.02	1.56 ± 0.77	3.00 ± 1.58
		hydro + 0.2 PEG	0.13 ± 0.00	0.21 ± 0.03	0.83 ± 0.14	1.23 ± 0.29
		hydro + 0.4 PEG	0.10 ± 0.01	0.09 ± 0.02	0.36 ± 0.05	0.46 ± 0.11
Sargodha 2002 White	osmotic stress	control	0.13 ± 0.02	0.22 ± 0.05	4.74 ± 1.40	13.4 ± 6.66
		0.2 PEG	0.17 ± 0.01	0.32 ± 0.02	5.47 ± 0.31	9.08 ± 3.79
		0.4 PEG	0.17 ± 0.02	0.22 ± 0.02	6.29 ± 0.30	15.7 ± 1.78
	osmo-priming	1% chitosan	0.22 ± 0.01	0.25 ± 0.02	4.75 ± 0.44	16.1 ± 2.99
		1% chitosan + 0.2 PEG	0.23 ± 0.02	0.22 ± 0.05	6.78 ± 1.60	21.6 ± 7.31
		1% chitosan + 0.4 PEG	0.26 ± 0.02	0.26 ± 0.02	6.41 ± 1.50	26.6 ± 9.63
	osmo-priming	2% chitosan	0.26 ± 0.01	0.25 ± 0.01	5.18 ± 0.51	20.5 ± 3.31
		2% chitosan + 0.2 PEG	0.26 ± 0.01	0.19 ± 0.01	6.00 ± 0.83	18.0 ± 4.42
		2% chitosan + 0.4 PEG	0.27 ± 0.03	0.22 ± 0.02	5.87 ± 0.49	19.1 ± 3.20
	thermo-priming	4 °C	0.17 ± 0.01	0.22 ± 0.03	4.92 ± 0.58	13.5 ± 1.95
		4 °C + 0.2 PEG	0.19 ± 0.02	0.18 ± 0.02	5.87 ± 0.54	18.4 ± 4.64
		4 °C + 0.4 PEG	0.22 ± 0.02	0.19 ± 0.02	7.27 ± 0.86	24.0 ± 8.72
	hydro-priming	hydro	0.18 ± 0.04	0.18 ± 0.03	5.14 ± 0.44	16.2 ± 1.07
		hydro + 0.2 PEG	0.20 ± 0.02	0.24 ± 0.01	7.35 ± 2.02	26.8 ± 8.32
		hydro + 0.4 PEG	0.16 ± 0.01	0.18 ± 0.01	5.67 ± 0.67	15.8 ± 2.78

a AGR = absolute growth rate, RGR = relative growth rate, NAR = net assimilation rate.

**Table 2:** Effect of Osmo-Priming, Thermo-Priming, and Hydro-Priming on Crop Growth Rate, Leaf Area Ratio, Root Shoot Ratio, and Percent Moisture Contents under Polyethylene Glycol (PEG)-Induced Osmotic Stress^a^

variety	treatment		CGR	LAR	RSR	PMC
Pearl	osmotic stress	control	0.02 ± 0.00	288.23 ± 18.86	9.02 ± 44.04	7.37 ± 1.29
		0.2 PEG	0.04 ± 0.00	243.0 ± 19.1	2.68 ± 0.68	8.88 ± 1.13
		0.4 PEG	0.06 ± 0.02	236.7 ± 97.2	3.14 ± 2.25	8.92 ± 2.53
	osmo-priming	1% chitosan	0.12 ± 0.02	101.4 ± 11.4	0.46 ± 0.09	4.71 ± 0.36
		1% chitosan + 0.2 PEG	0.13 ± 0.00	102.7 ± 1.92	0.34 ± 0.13	5.09 ± 0.34
		1% chitosan + 0.4 PEG	0.04 ± 0.01	227.4 ± 54.4	2.02 ± 1.62	9.97 ± 1.54
	osmo-priming	2% chitosan	0.03 ± 0.01	264.7 ± 46.6	0.82 ± 0.14	14.23 ± 2.2
		2% chitosan + 0.2 PEG	0.05 ± 0.02	234.7 ± 70.5	0.78 ± 0.48	8.54 ± 1.90
		2% chitosan + 0.4 PEG	0.08 ± 0.03	188.9 ± 69.7	0.55 ± 0.26	9.74 ± 3.76
	thermo-priming	4 °C	0.04 ± 0.01	225.6 ± 36.9	0.56 ± 0.18	8.75 ± 0.50
		4 °C + 0.2 PEG	0.03 ± 0.00	243.4 ± 38.0	0.55 ± 0.14	10.23 ± 1.2
		4 °C + 0.4 PEG	0.06 ± 0.01	169.0 ± 20.1	0.25 ± 0.03	7.78 ± 0.51
	hydro-priming	hydro	0.04 ± 0.02	340.30 ± 102	1.16 ± 0.45	12.13 ± 2.8
		hydro + 0.2 PEG	0.02 ± 0.00	317.2 ± 39.5	0.73 ± 0.10	14.11 ± 2.2
		hydro + 0.4 PEG	0.01 ± 0.00	610.3 ± 47.8	1.95 ± 0.23	23.48 ± 3.2
Sargodha 2002 White	osmotic stress	control	0.11 ± 0.03	78.73 ± 12.8	0.15 ± 0.01	2.75 ± 0.07
		0.2 PEG	0.13 ± 0.01	63.09 ± 7.07	0.12 ± 0.01	2.70 ± 0.25
		0.4 PEG	0.15 ± 0.01	63.03 ± 2.68	0.06 ± 0.00	2.64 ± 0.17
	osmo-priming	1% chitosan	0.11 ± 0.01	84.68 ± 6.70	0.12 ± 0.02	3.21 ± 0.07
		1% chitosan + 0.2 PEG	0.16 ± 0.04	61.44 ± 7.92	0.14 ± 0.02	2.80 ± 0.36
		1% chitosan + 0.4 PEG	0.15 ± 0.04	73.07 ± 13.4	0.23 ± 0.04	3.15 ± 0.04
	osmo-priming	2% chitosan	0.12 ± 0.01	83.80 ± 4.24	0.14 ± 0.01	3.08 ± 0.14
		2% chitosan + 0.2 PEG	0.14 ± 0.02	63.76 ± 4.78	0.20 ± 0.02	2.86 ± 0.06
		2% chitosan + 0.4 PEG	0.14 ± 0.01	68.11 ± 2.66	0.17 ± 0.02	2.50 ± 0.06
	thermo-priming	4 °C	0.12 ± 0.01	75.98 ± 7.33	0.17 ± 0.01	2.98 ± 0.11
		4 °C + 0.2 PEG	0.14 ± 0.01	74.22 ± 3.00	0.17 ± 0.01	3.23 ± 0.19
		4 °C + 0.4 PEG	0.17 ± 0.02	52.90 ± 2.32	0.15 ± 0.01	2.76 ± 0.15
	hydro-priming	hydro	0.12 ± 0.01	81.57 ± 7.50	0.17 ± 0.03	2.98 ± 0.24
		hydro + 0.2 PEG	0.18 ± 0.05	69.76 ± 13.3	0.15 ± 0.04	2.99 ± 0.12
		hydro + 0.4 PEG	0.14 ± 0.02	70.53 ± 5.21	0.16 ± 0.03	3.07 ± 0.07

a CGR = crop growth rate, LAR = leaf area ratio, RSR = root shoot ratio, PMC = percent moisture content.

**Table 3:** Effect of Osmo-Priming, Thermo-Priming, and Hydro-Priming on Final Germination Percentage, Seed Vigor Index, Coefficient of Velocity of Germination, and Timson Germination Index under Polyethylene Glycol (PEG)-Induced Osmotic Stress^a^

variety	treatment		FEP	SVI-1	SVI-2	CVG
Pearl	osmotic stress	control	73.33 ± 2.98	835.94 ± 38.09	9.080 ± 5.79	1.50 ± 0.13
		0.2 PEG	46.67 ± 2.98	633.78 ± 77.58	13.92 ± 3.16	0.70 ± 0.08
		0.4 PEG	53.33 ± 2.98	902.61 ± 102.6	37.2 ± 18.01	0.62 ± 0.11
	osmo-priming	1% chitosan	43.33 ± 2.98	776.67 ± 49.12	63.0 ± 10.32	0.58 ± 0.10
		1% chitosan + 0.2 PEG	53.33 ± 2.98	1016.39 ± 85.6	97.00 ± 6.39	0.95 ± 0.04
		1% chitosan + 0.4 PEG	56.67 ± 5.96	931.78 ± 158.1	25.5 ± 11.64	1.19 ± 0.10
	osmo-priming	2% chitosan	66.67 ± 2.98	1096.11 ± 92.3	19.45 ± 5.73	1.06 ± 0.16
		2% chitosan + 0.2 PEG	70.00 ± 5.16	970.94 ± 53.07	42.8 ± 16.84	1.18 ± 0.13
		2% chitosan + 0.4 PEG	76.67 ± 2.98	1501.3 ± 132.7	77.1 ± 30.12	1.42 ± 0.06
	thermo-priming	4 °C	63.33 ± 2.98	855.67 ± 150.0	27.530 ± .96	0.97 ± 0.07
		4 °C + 0.2 PEG	66.67 ± 2.98	1029.28 ± 19.0	25.6 ± ±4.89	1.01 ± 0.10
		4 °C + 0.4 PEG	63.33 ± 2.98	996.94 ± 54.98	53.1 ± 10.05	1.15 ± 0.03
	hydro-priming	hydro	70.00 ± 5.16	836.72 ± 128.4	35.5 ± 27.20	0.90 ± 0.06
		hydro + 0.2 PEG	66.67 ± 2.98	868.11 ± 127.4	9.480 ± 1.50	1.25 ± 0.14
		hydro + 0.4 PEG	66.67 ± 2.98	845.94 ± 103.7	4.430 ± 0.10	1.35 ± 0.03
Sargoda 2002 White	osmotic stress	control	63.33 ± 2.98	908.06 ± 104.1	116.7 ± 34.0	1.18 ± 0.05
		0.2 PEG	60.00 ± 0.00	953.33 ± 12.92	123.0 ± 8.63	1.23 ± 0.10
		0.4 PEG	60.00 ± 0.00	1036.33 ± 105.	150.7 ± 6.63	1.11 ± 0.05
	osmo-priming	1% chitosan	63.33 ± 2.98	1084.33 ± 89.1	115.3 ± 12.8	1.33 ± 0.01
		1% chitosan + 0.2 PEG	63.33 ± 2.98	1150.33 ± 95.9	175.6 ± 46.8	1.20 ± 0.12
		1% chitosan + 0.4 PEG	63.33 ± 2.98	1215.06 ± 41.3	155.7 ± 35.9	1.08 ± 0.05
	osmo-priming	2% chitosan	60.00 ± 0.00	1030.00 ± 84.6	120.1 ± 13.7	1.18 ± 0.05
		2% chitosan + 0.2 PEG	63.33 ± 2.98	1033.61 ± 71.3	150.5 ± 17.4	1.16 ± 0.05
		2% chitosan + 0.4 PEG	66.67 ± 2.98	1123.39 ± 37.9	157.1 ± 19.3	1.12 ± 0.07
	thermo-priming	4 °C	56.67 ± 2.98	862.33 ± 53.09	109.6 ± 17.6	1.19 ± 0.04
		4 °C + 0.2 PEG	70.00 ± 0.00	1237.06 ± 98.2	165.6 ± 15.1	1.50 ± 0.06
		4 °C + 0.4 PEG	66.67 ± 2.98	1228.33 ± 26.9	195.1 ± 14.7	1.51 ± 0.04
	hydro-priming	hydro	56.67 ± 2.98	854.44 ± 60.74	115.0 ± 4.47	1.34 ± 0.12
		hydro + 0.2 PEG	63.33 ± 2.98	1031.06 ± 16.6	183.5 ± 49.6	1.35 ± 0.07
		hydro + 0.4 PEG	63.33 ± 2.98	1071.89 ± 49.7	146.6 ± 25.7	1.44 ± 0.12

a FEP = final emergence percentage, SVI = seed vigor index, CVG = coefficient of velocity of germination.

**Table 4:** Effect of Osmo-Priming, Thermo-Priming, and Hydro-Priming on Leaf Area Index, Mean Emergence Time, and Timson Germination Index under Polyethylene Glycol (PEG)-Induced Osmotic Stress^a^

variety	treatment		LAI	MET	TGI
Pearl	osmotic stress	control	6.97 ± 0.15	0.39 ± 0.02	2.55 ± 0.12
		0.2 PEG	9.98 ± 0.15	0.58 ± 0.04	1.74 ± 0.11
		0.4 PEG	8.69 ± 1.21	0.65 ± 0.06	1.57 ± 0.16
	osmo-priming	1% chitosan	11.97 ± 1.20	0.66 ± 0.06	1.55 ± 0.15
		1% chitosan + 0.2 PEG	13.82 ± 0.52	0.50 ± 0.01	2.02 ± 0.06
		1% chitosan + 0.4 PEG	7.93 ± 1.19	0.43 ± 0.01	2.31 ± 0.08
	osmo-priming	2% chitosan	6.87 ± 1.22	0.48 ± 0.04	2.12 ± 0.17
		2% chitosan + 0.2 PEG	8.98 ± 1.34	0.45 ± 0.03	2.24 ± 0.15
		2% chitosan + 0.4 PEG	9.84 ± 2.39	0.40 ± 0.01	2.48 ± 0.04
	thermo-priming	4 °C	7.97 ± 0.59	0.50 ± 0.01	2.02 ± 0.06
		4 °C + 0.2 PEG	8.45 ± 0.35	0.49 ± 0.03	2.07 ± 0.11
		4 °C + 0.4 PEG	10.27 ± 0.53	0.45 ± 0.01	2.24 ± 0.04
	hydro-priming	hydro	8.48 ± 0.71	0.51 ± 0.02	1.95 ± 0.08
		hydro + 0.2 PEG	6.18 ± 0.53	0.43 ± 0.03	2.33 ± 0.15
		hydro + 0.4 PEG	5.34 ± 0.39	0.41 ± 0.01	2.43 ± 0.04
Sargodha 2002 White	osmotic stress	control	8.39 ± 2.04	0.44 ± 0.01	2.29 ± 0.07
		0.2 PEG	8.65 ± 1.28	0.43 ± 0.02	2.33 ± 0.09
		0.4 PEG	9.85 ± 0.76	0.46 ± 0.02	2.19 ± 0.06
	osmo-priming	1% chitosan	9.75 ± 0.30	0.42 ± 0.00	2.40 ± 0.02
		1% chitosan + 0.2 PEG	9.54 ± 1.52	0.44 ± 0.02	2.29 ± 0.13
		1% chitosan + 0.4 PEG	10.36 ± 1.14	0.47 ± 0.01	2.14 ± 0.06
	osmo-priming	2% chitosan	10.60 ± 0.88	0.44 ± 0.01	2.26 ± 0.08
		2% chitosan + 0.2 PEG	9.35 ± 1.31	0.45 ± 0.01	2.24 ± 0.06
		2% chitosan + 0.4 PEG	9.91 ± 1.01	0.45 ± 0.01	2.21 ± 0.06
	thermo-priming	4 °C	9.11 ± 0.89	0.44 ± 0.01	2.29 ± 0.07
		4 °C + 0.2 PEG	10.78 ± 1.10	0.39 ± 0.01	2.57 ± 0.06
		4 °C + 0.4 PEG	9.51 ± 1.28	0.39 ± 0.01	2.57 ± 0.04
	hydro-priming	hydro	10.16 ± 0.12	0.41 ± 0.02	2.45 ± 0.14
		hydro + 0.2 PEG	11.09 ± 0.75	0.41 ± 0.01	2.43 ± 0.07
		hydro + 0.4 PEG	9.93 ± 1.39	0.40 ± 0.02	2.52 ± 0.11

a LAI = leaf area index, MET = mean emergence time, TGI = Timson germination index.

According to the results, significant effects were found in variety × treatment (Table 5). AGR (absolute growth rate) values were recorded as 0.093 and 0.06 with a significant level (*p* < 0.001), AGR in osmotic stress treatment was 0.566 (*p* < 0.001), and treatment × variety was at 0.36 (*p* < 0.0036). RGRs (relative growth rates) were reported to be 64.126 and 53.683 (*p* < 0.0426). LAI (leaf area index) values over treatment were not significant but were having a significant difference in variety × treatment (*p* < 0.0204). Osmotic stressed and seed priming with chitosan were found to be significant in terms of CGR (crop growth rate) (*p* < 0.0461); likewise, the LAR (leaf area ratio) was highly significant (*p* < 0.0001), PMC (percent moisture content) shows a high level of significance (*p* < 0.001), MET (mean emergence time) showed significant values (*p* < 0.0001), SVI (seed vigor index) showed non-significant values (*p* < 0.0551), and the values of CVG (coefficient of velocity of germination) and TGI (Timson germination index) showed a significant difference (*p* < 0.001).

**Table 5:** Analysis of Variance of Measured Traits under Polyethylene Glycol-Induced Osmotic Stress in *Z. mays* L.^a^

trait	source	SS	Df	MS	F	P
AGR-1	treatment	0.093	14	0.007	5.9709	0.0000***
	variety	0.06	1	0.06	54.234	0.0000***
	treatment × variety	0.049	14	0.003	3.1252	0.0011***
	error	0.067	60	0.001		
AGR-2	treatment	0.566	14	0.04	4.2837	0.0000***
	variety	0.191	1	0.191	20.292	0.0000***
	treatment × variety	0.36	14	0.026	2.7255	0.0036**
	error	0.566	60	0.009		
RGR	treatment	64.126	14	4.58	1.9142	0.0426**
	variety	312.966	1	312.966	130.78	0.0000***
	treatment × variety	53.683	14	3.834	1.6024	0.1048
	error	143.574	60	2.393		
LAI	treatment	93.911	14	6.708	1.5339	0.1268
	variety	23.226	1	23.226	5.3110	0.0247**
	treatment × variety	132.308	14	9.45	2.1609	0.0204**
	error	262.389	60	4.373		
CGR	treatment	0.036	14	0.003	1.8836	0.0467**
	variety	0.18	1	0.18	130.86	0.0000***
	treatment × variety	0.03	14	0.002	1.5797	0.1117
	error	0.083	60	0.001		
LAR	treatment	30,8190.9	14	22013.63	4.0364	0.0001***
	variety	744,962.4	1	744962.4	136.59	0.0000***
	treatment × variety	294,442.7	14	21031.62	3.8563	0.0001***
	error	327,227.1	60	5453.785		
RSR	treatment	102.923	14	7.352	2.3353	0.012**
	variety	51.68	1	51.68	16.416	0.0001***
	treatment × variety	104.061	14	7.433	2.3611	0.0111**
	error	188.885	60	3.148		
PMC	treatment	447.23	14	31.945	4.1842	0.0000***
	variety	1215.286	1	1215.286	159.17	0.0000***
	treatment × variety	425.166	14	30.369	3.9778	0.0001***
	error	458.082	60	7.635		
MET	treatment	0.161	14	0.012	5.1354	0.0000***
	variety	0.084	1	0.084	37.475	0.0000***
	treatment × variety	0.158	14	0.011	5.0372	0.0000***
	error	0.135	60	0.002		
FEP	treatment	2528.889	14	180.635	0.9264	0.0000***
	variety	1.111	1	1.111	0.0303	0.0000***
	treatment × variety	1982.222	14	141.5873	3.8615	0.0001***
	error	2200	60	36.667		
SVI-1	treatment	1,541,522	14	110108.7	3.9055	0.0001***
	variety	296,296.5	1	296296.5	50210.1	0.0019***
	treatment × variety	669,494.8	14	47821.06	1.6962	0.0804
	error	1,691,575	60	28192.92		
SVI-2	treatment	40,003.51	14	2857.394	1.8274	0.0551*
	variety	268,768.7	1	268768.7	171.88	0.0000***
	treatment × variety	21,746.3	14	1553.307	0.9934	0.0000***
	error	93,818.6	60	1563.643		
CVG	treatment	2.033	14	0.145	5.136	0.0000***
	variety	0.961	1	0.961	33.981	0.0000***
	treatment × variety	2.04	14	0.146	5.1527	0.0000***
	error	1.697	60	0.028		
TGI	treatment	2.443	14	0.175	4.8614	0.0000***
	variety	1.265	1	1.265	35.236	0.0000***
	treatment × variety	2.344	14	0.167	4.6639	0.0000***
	error	2.154	60	0.036		

a AGR = absolute growth rate, RGR = relative growth rate, LAI = leaf area index, CGR = crop growth rate, LAR = leaf area ratio, RSR = root shoot ratio, PMC = percent moisture content, MET = mean emergence time, FEP = final emergence percentage, SVI = seed vigor index, CVG = coefficient of velocity of germination, TGI = Timson germination index. * is significant up to *p* = 0.05, ** is significant at *p* = 0.01, and *** is significant at *p* = 0.001.

### 3.2 Physiological and Biochemical Attributes

#### 3.2.1 Photosynthetic Pigments (Chlorophyll a and b and Carotenoids)

**Figure 3:**
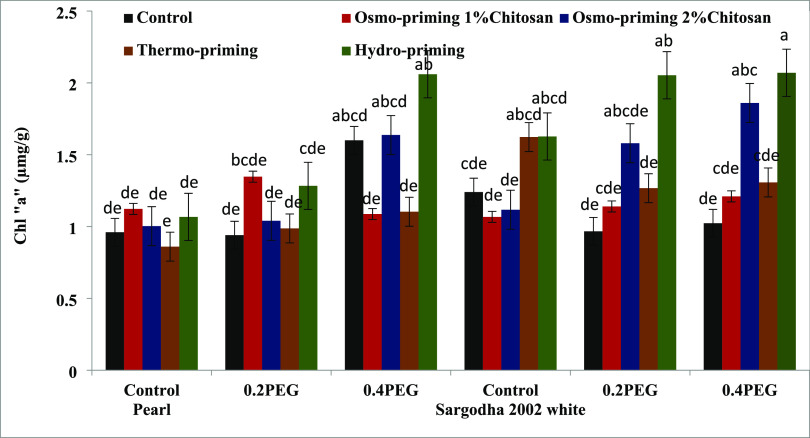
Effect of osmo-priming, thermo-priming, and hydro-priming on the chlorophyll “a” content of *Z. mays* L. under polyethylene glycol-induced osmotic stress (mean ± standard error). Letters indicating least significant difference among the mean values at *p* ≤ 0.05.

**Figure 4:**
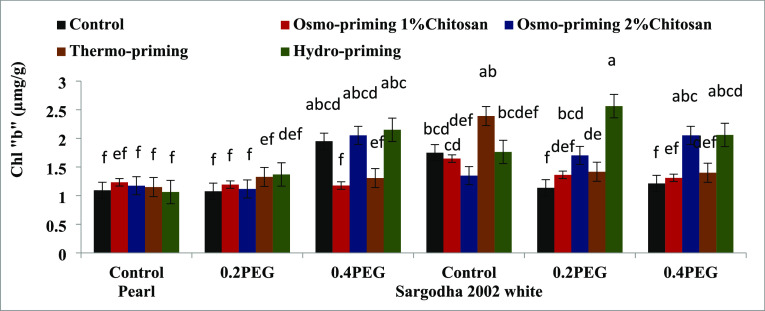
Effect of osmo-priming, thermo-priming, and hydro-priming on the chlorophyll “b” content of *Z. mays* L. under polyethylene glycol-induced osmotic stress (mean ± standard error). Letters indicating least significant difference among the mean values at *p* ≤ 0.05.

**Figure 5:**
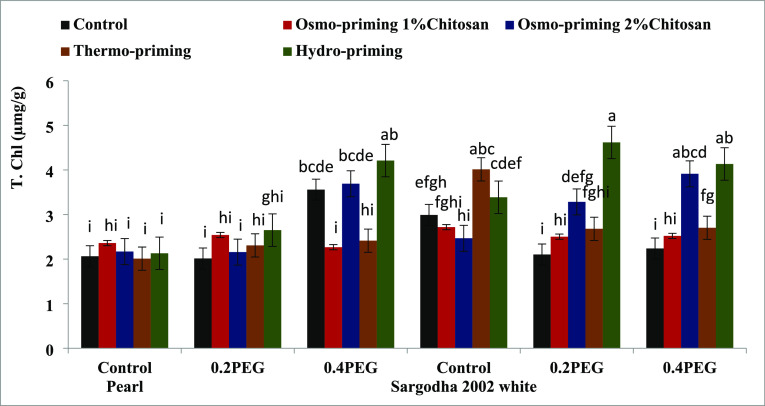
Effect of osmo-priming, thermo-priming, and hydro-priming on the total chlorophyll content of *Z. mays* L. under polyethylene glycol-induced osmotic stress (mean ± standard error). Letters indicating least significant difference among the mean values at *p* ≤ 0.05.

**Figure 6:**
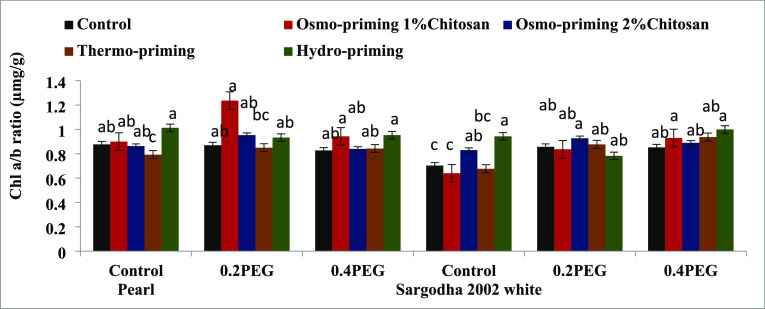
Effect of osmo-priming, thermo-priming, and hydro-priming on the chlorophyll a/b ratio content of *Z. mays* L. under polyethylene glycol-induced osmotic stress (mean ± standard error). Letters indicating least significant difference among the mean values at *p* ≤ 0.05.

**Figure 7:**
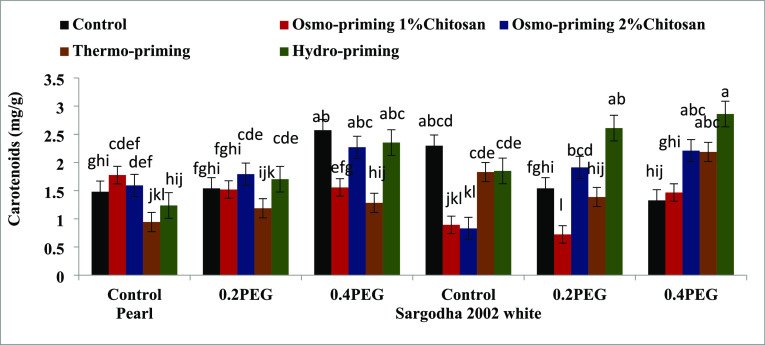
Effect of osmo-priming, thermo-priming, and hydro-priming on the total carotenoid content of *Z. mays* L. under polyethylene glycol-induced osmotic stress (mean ± standard error). Letters indicating least significant difference among the mean values at *p* ≤ 0.05.

To monitor the plant stress, we need an accurate estimation of the chlorophyll content, which is different in different plant species. The results indicated that the concentrations of the chlorophyll and carotenoid content of *Z. mays* L. were affected under induced polyethylene glycol (PEG) stress (Figures 3–7). Osmo-priming with chitosan and hydro-priming with water increased the chlorophyll and carotenoid content. The chlorophyll content was decreased under stress conditions. On the contrary, it was reported that hydro-priming treatment under 0.4 MPa induced osmotic stress of polyethylene glycol and the chlorophyll and carotenoid content were increased. After hydro-priming, osmo-priming with chitosan (2%) showed the maximum value of the chlorophyll content. Results suggested that osmotic stress provided in the form of polyethylene glycol (PEG) reduced the growth responses by decreasing the chlorophyll content; osmo-priming with chitosan can adjust ion homeostasis caused by PEG.

#### 3.2.2 Total Sugar Content

**Figure 8:**
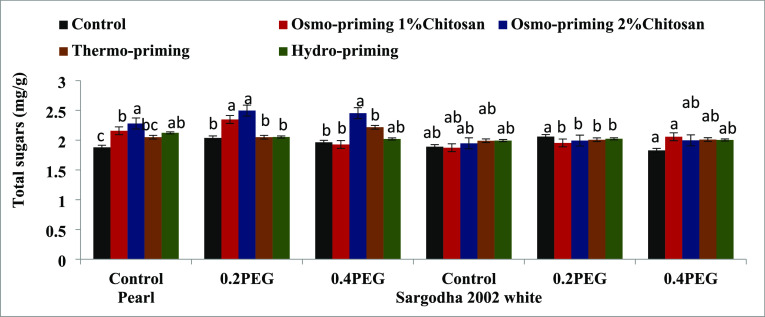
Effect of osmo-priming, thermo-priming, and hydro-priming on the total sugar content of *Z. mays* L. under polyethylene glycol-induced osmotic stress (mean ± standard error). Letters indicating least significant difference among the mean values at *p* ≤ 0.05.

The total sugar content of *Z. mays* L. subjected to osmo-priming with chitosan (1 and 2%), thermo-priming at 4 °C, and hydro-priming with water under polyethylene glycol (PEG)-induced osmotic stress was found to be non-significant (*p* < 0.05) in both the varieties. Osmo-priming with chitosan 2% was effective in terms of the total sugar content (Figure 8).

#### 3.2.3 Total Protein Content

**Figure 9:**
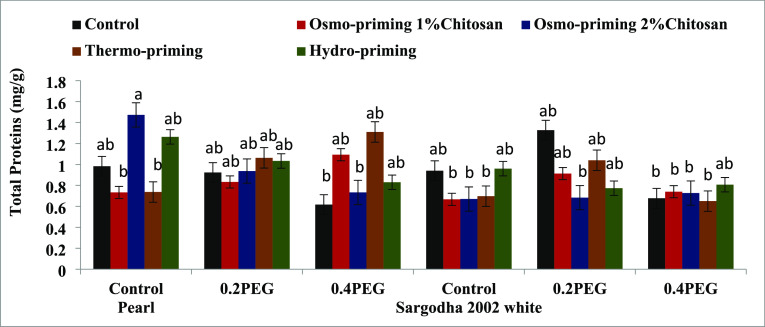
Effect of osmo-priming, thermo-priming, and hydro-priming on the total protein content of *Z. mays* L. under polyethylene glycol-induced osmotic stress (mean ± standard error). Letters indicating least significant difference among the mean values at *p* ≤ 0.05.

The total protein content of *Z. mays* L. subjected to varying levels of osmotic stress with applied treatments, concentration, and their interactive effect showed significant differences (*p* < 0.05). The maximum value of protein was reported in control of osmo-priming with chitosan 2% in pearl variety followed by thermo-priming (Figure 9).

#### 3.2.4 Total Proline Content

**Figure 10:**
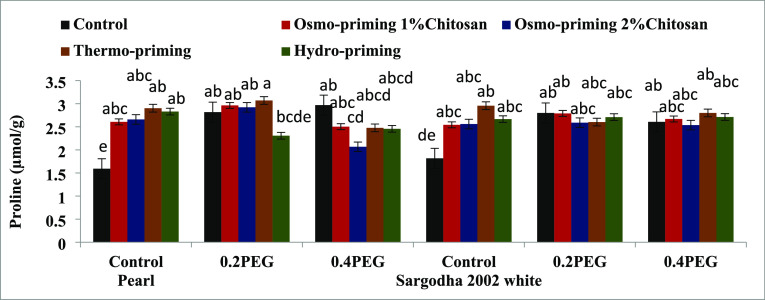
Effect of osmo-priming, thermo-priming, and hydro-priming on the total proline content of *Z. mays* L. under polyethylene glycol-induced osmotic stress (mean ± standard error). Letters indicating least significant difference among the mean values at *p* ≤ 0.05.

The total proline content of *Z. mays* L. subjected to osmotic stress with applied treatment and concentrations showed significant differences (*p* < 0.05). The maximum value was reported in thermo-priming of pearl variety. The interactive effect of treatment into the concentration also showed significant differences (*p* < 0.05). Thermo-priming was effective under 0.2 MPa of induced polyethylene glycol osmotic stress (Figure 10).

#### 3.2.5 Antioxidant Enzymes (SOD, POD, APX, and CAT)

**Figure 11:**
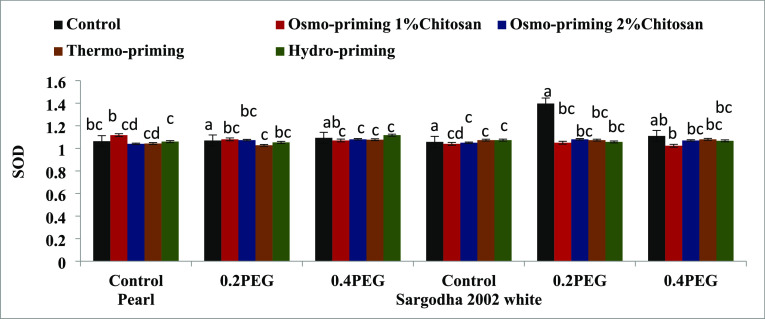
Effect of osmo-priming, thermo-priming, and hydro-priming on the superoxide dismutase enzyme (SOD) content of *Z. mays* L. under polyethylene glycol-induced osmotic stress (mean ± standard error). Letters indicating least significant difference among the mean values at *p* ≤ 0.05.

**Figure 12:**
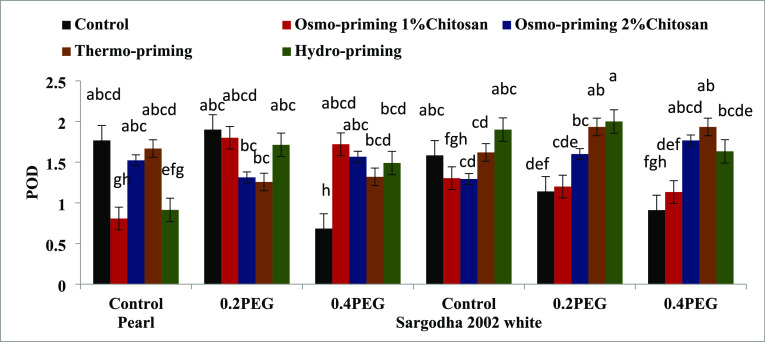
Effect of osmo-priming, thermo-priming, and hydro-priming on the peroxidase enzyme (POD) content of *Z. mays* L. under polyethylene glycol-induced osmotic stress (mean ± standard error). Letters (a–h) indicating least significant difference among the mean values at *p* ≤ 0.05.

The superoxide dismutase (SOD) enzyme content of *Z. mays* L. subjected to osmo-priming with chitosan (1 and 2%), thermo-priming at 4 °C, and hydro-priming under polyethylene glycol (PEG)-induced osmotic stress showed non-significant differences (*p* < 0.05) (Figure 11). Peroxidase (POD) enzyme activity of *Z. mays* L. subjected with chitosan osmo-priming (1 and 2%), thermo-priming at 4 °C, and hydro-priming under polyethylene glycol (PEG)-induced osmotic stress was significant (*p* < 0.05). The maximum value of POD was found with hydro-priming in both the varieties followed by osmo-priming with chitosan 1% (Figure 12).

**Figure 13:**
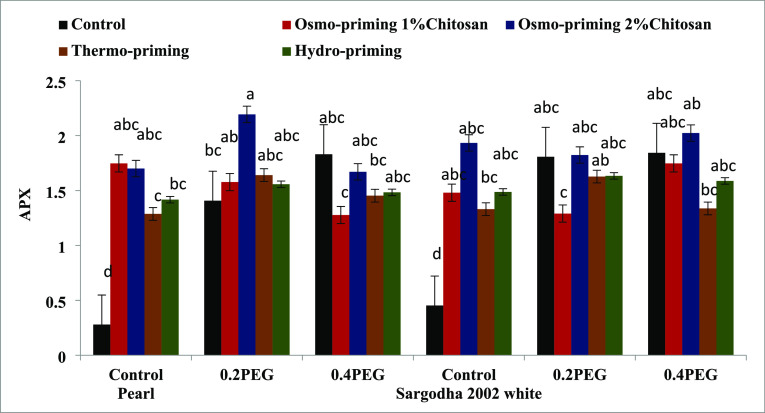
Effect of osmo-priming, thermo-priming, and hydro-priming on the ascorbate peroxidase enzyme (APX) content of *Z. mays* L. under polyethylene glycol-induced osmotic stress (mean ± standard error). Letters indicating least significant difference among the mean values at *p* ≤ 0.05.

**Figure 14:**
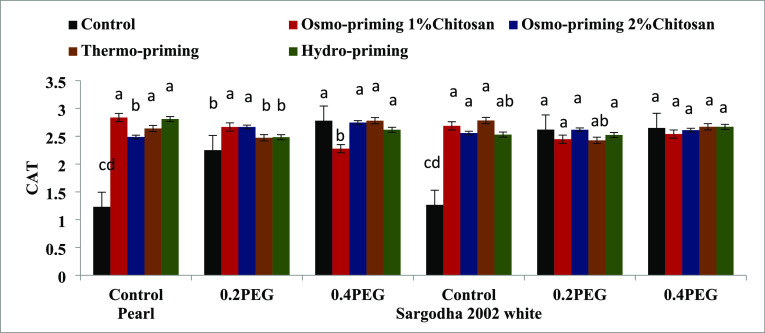
Effect of osmo-priming, thermo-priming, and hydro-priming on the catalase enzyme (CAT) content of *Z. mays* L. under polyethylene glycol-induced osmotic stress (mean ± standard error). Letters indicating least significant difference among the mean values at *p* ≤ 0.05.

The ascorbate peroxidase (APX) enzyme content under osmo-priming with chitosan (1 and 2%), thermo-priming at 4 °C, and hydro-priming with water under polyethylene glycol (PEG)-induced osmotic stress showed a significant increase (*p* < 0.05). The maximum level of APX was found in osmo-priming with chitosan 2% in the controlled group as well as in stressed conditions in both the varieties followed by osmo-priming with chitosan 1% (Figure 13). Activity of the catalase (CAT) enzyme content indicated a significant increase (*p* < 0.05) as the maximum concentration was reported in osmo-priming with chitosan 1% followed by osmo-priming with chitosan 2%. The interactive effects of treatment, concentration, and variety were found to be non-significant at *p* < 0.05 (Figure 14).

### 3.3 Analysis of Variance of Measured Trait under Polyethylene-Induced Osmotic Stress in *Z. mays* L.

Analysis of variance (ANOVA) for chlorophyll a, chlorophyll b, and total chlorophyll content revealed significant differences (*p* < 0.05) in treatment (A), concentration (B), and variety (C). Moreover, the interactive effect of treatment and concentration (AxB) and treatment and variety (AxC) was significant (*p* < 0.05) in chlorophyll b and the concentration and variety were found to be significant (*p* < 0.05) in chlorophyll b and total chlorophyll content, under polyethylene glycol-induced osmotic stress (Table 6).

**Table 6:** Mean Square of the ANOVA for Chlorophyll a, Chlorophyll b, and Total Chlorophyll Content of *Z. mays* L. under Polyethylene Glycol-Induced Osmotic Stress^a^

sources	degree of freedom	chlorophyll a	chlorophyll b	total chlorophyll content
treatment (A)	4	0.999*	0.724*	3.347*
concentration (B)	2	0.854*	0.508^NS^	2.586*
AxB	8	0.226^NS^	0.527*	1.393^NS^
variety (C)	1	0.928*	2.196^NS^	5.975*
AxC	4	0.295^NS^	0.248*	1.024^NS^
BxC	2	0.246^NS^	1.14*	2.444*
AxBxC	8	0.132^NS^	0.263^NS^	0.736^NS^
error	60	0.195	0.207	0.711
coefficient of variance (%)		33.79%	29.94%	29.83%

a * = significant (*p* < 0.05), NS = non-significant (*p* > 0.05).

Chlorophyll ratio a/b, total carotenoid contents, and total sugar contents revealed significant differences (*p* < 0.05) in treatment (A), concentration (B), and variety (C). Moreover, the interactive effect of treatment and variety was also found to be significant (*p* < 0.05) in terms of the total carotenoid content and total sugar contents. Total sugar contents show high significant values, i.e., (*p* < 0.001) in terms of variety (C) (Table 7).

**Table 7:** Mean Square of the ANOVA for Chlorophyll Ratio “a/b”, Total Carotenoid Content, and Total Sugar Content of *Z. mays* L. under Polyethylene-Induced Osmotic Stress^a^

sources	degree of freedom	chlorophyll ratio “a/b”	total carotenoid content	total sugar content
treatment (A)	4	0.043^NS^	1.666*	0.146*
concentration (B)	2	0.07^NS^	2.375*	0.054^NS^
AxB	8	0.033^NS^	0.452^NS^	0.022^NS^
variety (C)	1	0.103^NS^	0.124^NS^	0.59***
AxC	4	0.038^NS^	1.454*	0.116*
BxC	2	0.066^NS^	0.033^NS^	0.005^NS^
AxBxC	8	0.011^NS^	0.626^NS^	0.038^NS^
error	60	0.047	0.371	0.027
coefficient of variance (%)		33.79%	24.58%	36.00%

a * = significant (*p* < 0.05), NS = non-significant (*p* > 0.05).

The total proline content revealed significant differences (*p* < 0.05) in treatment (A) and concentration (B). Moreover, the interactive effect of treatment and concentration (AxB) in terms of the total proline content showed significant differences (*p* < 0.0001). The total protein content and antioxidant enzyme superoxide dismutase were non-significant (Table 8).

**Table 8:** Mean Square of the ANOVA of the Total Protein Content, Total Proline Content, and Superoxide Dismutase Enzyme of *Z. mays* L. under Polyethylene-Induced Osmotic Stress^a^

sources	degree of freedom	total protein content	total proline content	superoxide dismutase
treatment (A)	4	0.036^NS^	0.339*	0.016^NS^
concentration (B)	2	0.143^NS^	0.476*	0.009^NS^
AxB	8	0.202^NS^	0.659***	0.012^NS^
variety (C)	1	0.526*	0.005^NS^	0.005^NS^
AxC	4	0.155^NS^	0.035^NS^	0.017^NS^
BxC	2	0.119^NS^	0.159^NS^	0.017^NS^
AxBxC	8	0.129^NS^	0.161^NS^	0.01^NS^
error	60	0.116	0.1	0.012^NS^
coefficient of variance (%)		33.79%	38.08%	12.07%

a * = significant (*p* < 0.05), NS = non-significant (*p* > 0.05).

Activity of ascorbate peroxidase (APX) and catalase (CAT) revealed significant differences (*p* < 0.05) in treatment (A), concentration (B), and variety (C). Moreover, the interactive effect was also significant (*p* < 0.001) in terms of treatment and concentration (AxB) in these two antioxidant enzymes. The peroxidase enzyme showed significant differences (*p* < 0.05) only in the interactive effect of treatment and concentration (AxB), treatment and variety (AxC), and treatment, concentration, and variety (AxBxC) (Table 9).

**Table 9:** Means Square of the ANOVA of Peroxidase, Ascorbate Peroxidase, and Catalase Enzyme of *Z. mays* L. under Polyethylene Glycol-Induced Osmotic Stress^a^

sources	degree of freedom	peroxidase	ascorbate peroxidase	catalase
treatment (A)	4	0.374^NS^	0.922***	0.813***
concentration (B)	2	0.256^NS^	1.088***	0.475**
AxB	8	0.459*	0.744***	0.896***
variety (C)	1	0.228^NS^	0.078^NS^	0.002^NS^
AxC	4	0.519*	0.039^NS^	0.017^NS^
BxC	2	0.098^NS^	0.078^NS^	0.006^NS^
AxBxC	8	0.381*	0.123*	0.072^NS^
error	60	0.175	0.063	0.042
coefficient of variance (%)		33.79%	28.27%	16.42%

a * = significant (*p* < 0.05), NS = non-significant (*p* > 0.05).

### 3.4 Principle Component Analysis Based on the Correlation Matrix of Biological Components

**Figure 15:**
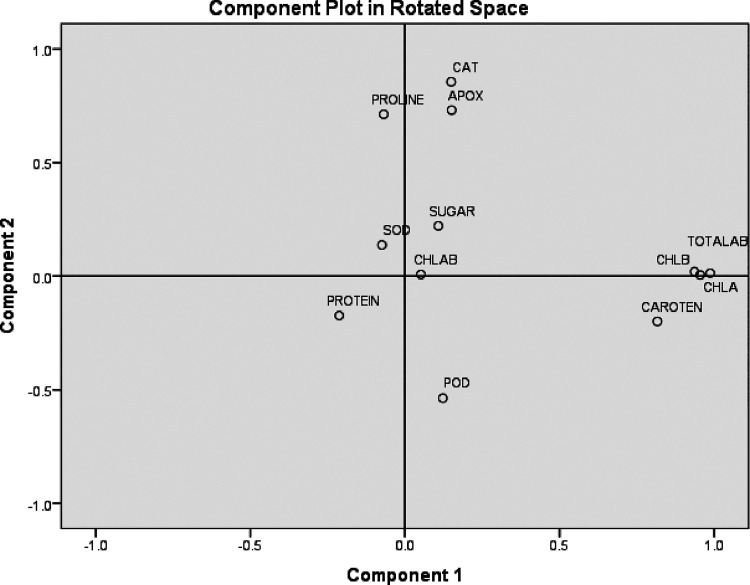
Component plot in the rotated space: principle component analysis (PCA) based on the correlation matrix of biological components*.*

The results of principle component analysis are based on 12 characters and represented that the first three components enclosed overall 59.753% of the total variation. The PC_1_ explained 29.910% of complete variance, which were significantly correlated with chlorophyll b, chlorophyll ratio, sugar, protein, and proline. The PC_1_ was particularly related to growth responses. The PC_2_ result explained 19.230% of the total variance, which was particularly correlated with chlorophyll a, total chlorophyll, chlorophyll a/b ratio, POD, SOD, and APX. The PC_2_ was related to the plant chlorophyll content and antioxidant enzymes. The PC_3_ was accounted with 10.613% of the whole variation, and the important variations under PC_3_ were carotenoids, soluble proteins, SOD, and APX enzymes. This indicated that PC_3_ was correlated to antioxidant enzymes and osmolytes. There were variations that were related with parameters among each other and were independent with variation of other components; consequently, we plotted three components in rotated space component design (Figure 15). There was a positive correlation found in PC_1_ and PC_2_, which included growth response and osmolytes. There was also a positive correlation found in PC_1_ and PC_3_, including growth response and antioxidant enzymes, respectively. There was no correlation found in PC_2_ and PC_3_ as we know that PC_2_ was related to growth response and PC_3_ was correlated with antioxidant enzymes (Table 10).

**Table 10:** Eigenvalues, Variation Explained (%), Cumulative Values (%), and Coefficient of Determination of the First Three Principle Components Based on the Correlation Matrix of Biological Components in *Z. mays* L.^a^

		variance %		components				
component	eigenvalues	individual	cumulative	PC_1_	PC_2_	PC_3_	PC_4_	PC_5_
chlorophyll a	3.589	29.910	29.910	0.959	0.050	0.089	0.072	0.172
chlorophyll b	2.308	19.230	49.139	0.910	0.106	0.255	0.040	0.239
total chlorophyll	1.274	10.613	59.753	0.975	0.083	0.096	0.057	0.046
chlorophyll ratio	1.177	9.810	69.563	0.103	0.118	0.613	0.036	0.751
carotenoids	1.134	9.447	79.010	0.816	0.135	0.222	0.128	0.001
sugar	0.766	6.386	85.396	0.158	0.396	0.640	0.021	0.367
protein	0.620	5.163	90.559	0.264	0.090	0.506	0.367	0.534
proline	0.542	4.514	95.073	0.026	0.753	0.063	0.300	0.037
SOD	0.323	2.693	97.766	0.109	0.190	0.124	0.814	0.162
POD	0.253	2.109	99.875	0.141	0.428	0.270	0.502	0.089
APX	0.015	0.125	100.000	0.208	0.745	0.070	0.019	0.160
CAT	2.876	2.396	100.000	0.211	0.865	0.049	0.104	0.013

a SOD = superoxide dismutase, POD = peroxidase, APX = ascorbate peroxidase, CAT = catalase.

## 4 Discussion

Osmotic stress affects plants during their life cycle, and seeds are mostly susceptible to these stresses between sowing and seedling establishment.^91^ The germination rate and seedling growth are influenced by nutritional imbalance and osmotic stress.^58^ The chitosan α,β-(1,4)-glucosamine polymer is a safe, natural, and cheap polysaccharide and is produced from chitin, which is the major structural component of the fungus cell wall and the exoskeleton of arthropods.^68,69^ Chitosan is used as plant fertilizer as it promotes seed germination and enhances germination percentage.^70^ Maize is the most important cereal crop and is grown all over the world.^92^ It is estimated that the demand of the maize crop will double in the developing countries.^93^ The present study was aimed at determining the effect of osmo-priming with chitosan (1 and 2%), thermo-priming at 4 °C, and hydro-priming on two varieties (Pearl and Sargodha 2002 White) of *Z. mays* L. under polyethylene glycol (PEG)-induced osmotic stress.

In the present study, the agronomic parameters were adversely affected by polyethylene glycol (PEG)-induced osmotic stress. The same results were reported in ref (94) in maize cultivar under (PEG)-induced osmotic stress. The seed priming technique proved to be useful (Table 1) that showed a significant difference in terms of absolute growth rate (AGR) under osmo-priming with chitosan 2% followed by hydro-priming. The relative growth rate (RGR) was also having a significant impact under osmo-priming with 1% chitosan with the highest value in pearl variety. Crop growth rate (CGR) was high under osmo-priming with 1% chitosan followed by hydro-priming in both the varieties. Similar results were reported in ref (95) in different cultivars of wheat under water deficit conditions. Osmo-priming, hydro-priming, and thermo-priming improved (AGR) and (RGR); hence, our findings were in agreement with the results of ref (96).

The percent moisture content (PMC) was influenced by the seed priming technique under induced osmotic stress. In hydro-priming, the PMC values were high in comparison with the controlled group. Osmo-priming with chitosan also shows PMC values greater than control conditions in the pearl variety of *Z. mays* L. under induced osmotic stress conditions. Osmotic stress reduced the PMC, thus confirming the results reported in ref (97) in *Z. mays* L. and *Gossypium hirsutum* L. under osmotic stress conditions. Chitosan promotes growth of cabbage (*Brassica oleracea* L. var. *Capitata* L.) callus *in vitro*,^98^ and chitin oligosaccharide increased the chitinase activity of rice.^99^ Chitosan priming on maize seed germination attributes and early seedling has a pronounced effect on growth under osmotic stress induced by PEG.^67^

Final germination percentage (FGP) was increased in osmo-priming with chitosan (2%) followed by controlled group, and parallel results were reported in ref (77) in wheat. They reported that chitosan priming has the potential to increase the FGP in wheat plant. Under PEG stress, chitosan as osmo-priming proved to be effective in terms of final germination percentage (FGP) and mean emergence time (MET) in wheat plant.^79^ This increase in seed germination rate with seed priming is due to certain biochemical changes like enzyme activation and metabolism or imbibition.^100^

Chitosan improves the germination of seeds under water deficit conditions in maize^76^ and in rice.^101^ Seed priming with chitosan showed a pronounced increase in seed germination percentage, activity of lipase enzyme, gibberellic acid, and indole acetic acid (IAA) levels in peanut.^70^ Additionally, seed priming probably permitted some repairs of membrane degradation damage and produced better germination patterns and higher vigor levels in comparison with unprimed seeds.^102^ In the present study, seeds primed with chitosan had better germination characteristics and seedlings grew more quickly when exposed to osmotic stress. It is clear that chitosan may be useful in high-stress situations. Application of chitosan enhances plant germination in drought-stressed regimes.^101^

The findings in Table 3 indicated that osmo-priming with chitosan (2%) increased the seed vigor index (SVI) in both the studied varieties of *Z. mays* L., and similar results were reported in ref (100) in *Carum copticum* plants, suggesting that with increasing chitosan concentration, the seed vigor index also improved. Chitosan is used as osmo-priming to decrease the adverse effects of oxidative stress. Chitosan is obtained by deacetylation of chitin; it promotes root and shoot growth in *Raphanus sativus* L. and accelerates flower timing and number in *Passiflora edulis.*(103) The vigor enhancement of maize seedlings with chitosan priming is reported in ref (104). Under drought stress regimes, cytokinesis and cell elongation are closely associated with the reduction in growth characteristics such as AGR, RGR, and MET. This decrease is thought to be caused by reduced photosynthesis and stomatal closure that ultimately causes the leaves to shrink.^104^ The low water content, reduced turgidity, wilting, closing of stomata, and eventually a decrease in cell expansion and growth are signs of a water deficit state. Plant growth is affected in different ways by both internal and external causes.^105^ It has been noted that in *Populus* species, root length decreased under osmotic stress.^106^ The primary cause of poor photosynthesis and low crop yield in water-scarce environments is reduced leaf area.^107^

Plants contain substantial amounts of carotenoids that serve as non-enzymatic scavengers of active oxygen species.^108^ Drought causes the chlorophyll breakdown and accelerates the leaf senescence.^109^ The concentrations of the chlorophyll and carotenoid content of *Z. mays* L. were adversely affected under induced polyethylene glycol PEG osmotic stress. Osmo-priming with chitosan and hydro-priming with water increased the chlorophyll and carotenoid content. Normally, under stress conditions, plants decrease the chlorophyll content.^110,111^ In the present study, chlorophyll a, chlorophyll b, total chlorophyll content, and chlorophyll ratio a/b were studied in both the varieties (Pearl and Sargodha 2002 White) of *Z. mays* L. under polyethylene glycol-induced osmotic stress. The chlorophyll content showed a marked decrease under water deficit conditions in maize. The chlorophyll content also decreased under osmotic stress conditions (0.2 and 0.4 MPa) as previously reported in ref (111). It was reported to be high in hydro-priming under 0.4 MPa of induced PEG osmotic stress. After hydro-priming, osmo-priming with chitosan (2%) showed maximum levels of the chlorophyll content. Similar results were reported in ref (112). The photosynthetic attributes of *Z. mays* L. were decreased under stress conditions.^113^ The seed priming technique with chitosan (1 and 2%) improved the chlorophyll content of *Z. maysL*. under stress conditions, and similar results were reported in ref (114) by studying the effects of foliar application of chitosan in maize and soybean (Figures 3–7).

Soluble solutes (sugar and protein content) mitigate the lethal effects of drought stress and maintain ionic balance in cells. The total soluble sugar content (Figure 8) significantly increased under osmo-priming with chitosan (2%), and chitosan improved the sugar content of both the varieties of maize, thus confirming the same findings made in ref (115). According to Wang *et al*.,^116^ it appears that the lesser amount of soluble sugars produced in response to melatonin-seed priming, particularly under drought stress, may be related to the ameliorative impact of melatonin on drought stress, which creates a favorable environment and prevents the plant from receiving any stress signals. In our study, there was no significant effect on the sugar content, but for osmo-priming with chitosan, the production of sugar increases non-significantly.

Proline production in the leaves is thought to play a significant role in the plant’s ability to respond to abiotic stress situations.^117,118^ The osmo-protective ability of proline under stress regimes is well recognized.^119^ Proline plays a critical part in the mechanism of osmotic adjustment in many crops under severely stressed conditions, and a rise in proline levels in plants during drought stress is thought to be a sign of drought stress resistance. Proline oxidase (PROX) and −glutamyl kinase (−GK), two significant enzymes, control the amount of proline in plants.^120^ The total proline content of *Z. mays* L. under induced PEG stress was studied, and the maximum value was reported in the controlled group of osmo-priming with chitosan (2%) followed by thermo-priming at 4 °C (Figure 10) in both the varieties. Chitosan improved the levels of the proline content, and parallel results were reported in ref (121) by studying the effect of chitosan under osmotic stress conditions in *Carthamus tinctorius* L. A marked spike was noted in the proline content when water deficit conditions dominated the plants.^112^ This increase can be correlated with the tolerance of plants under stress conditions.^122^

To combat ROS-induced cellular damage, plant cells have a sophisticated enzymatic antioxidant system.^123−125^ Non-enzymatic components like carotenoids, glutathione, and tocopherols work in conjunction with antioxidant enzymes such as superoxide dismutase (SOD), catalase (CAT), and glutathione peroxidase (GPX) in the elimination of ROS and other free radicals produced under osmotic stress regimes.^126^ Proteins, lipids, and nucleic acids are all attacked by ROS, and the extent of the damage relies on how well the antioxidative scavenging systems balance ROS generation.^127−130^ The maximum concentration of POD was found with hydro-priming in both the varieties followed by osmo-priming with chitosan 1%. Ascorbate peroxidase (APX) activity under osmo-priming with chitosan (1 and 2%), thermo-priming at 4 °C, and hydro-priming with water under polyethylene glycol (PEG)-induced osmotic stress showed a significant increase (*p* < 0.05). The maximum level of APX was found in osmo-priming with chitosan 2% in control as well as in stress conditions in both the varieties followed by osmo-priming with chitosan 1%. The catalase (CAT) content of *Z. mays* L. indicated a significant increase (*p* < 0.05) as the maximum concentration was reported in osmo-priming with chitosan 1% followed by osmo-priming with chitosan 2% (Figures 11–14). The plant antioxidant defense mechanism precisely regulates the balance between ROS generation and consumption when growth conditions are ideal.^131^ Chitosan contains certain anti-oxidative qualities and can serve as the ROS scavenger by boosting the antioxidant capacity of plant cells.^76^ Chitosan has been shown to boost the peroxidase activity in the roots of date palm, indicating that it is an exogenous inducer of defense responses.^132^ CAT, SOD, and POD activities increased significantly in the primed seeds, demonstrating a similar defense response caused by chitosan in this series of tests. In a prior study, it was found that chitosan treatments, either as a priming agent or growing media, resulted in enhanced CAT, POD, and SOD enzyme activity in low-temperature challenged maize.^76,133^ Upon fast desiccation, the embryonic axes of pedunculate oak (*Quercus robour*) seeds showed a considerable rise in POD activity and a concurrent decrease in total phenolic compounds. Based on these findings, they hypothesized that POD uses phenolic compounds as electron donors in the embryonic axis cells to scavenge H_2_O_2_. *Catanea sativa* embryonic axes under injury and desiccation have demonstrated a similar mechanism.^134^

## 5 Conclusions

In conclusion, it was evident that physiological and agronomic traits of both the varieties of *Z. mays* L. were affected adversely under polyethylene glycol-induced stress conditions. Osmo-priming with chitosan (1 and 2%), thermo-priming at 4 °C, and hydro-priming with distilled water proved to be effective in modulating agronomic and physiological attributes of maize cultivars under induced PEG osmotic stress. Moreover, activities of antioxidant enzymes including peroxidase (POD), superoxide dismutase (SOD), catalase (CAT), and ascorbate peroxidase (APX) were markedly improved under stress regimes with seed priming. In addition, osmo-priming with chitosan could be utilized to adjust the ionic differences of seeds in the soil under osmotic stress conditions. Importantly, in the present era of inevitable changing climatic shifts, fulfilling the increasing food demand of the rapidly expanding population is a daunting challenge for the scientific community as well as for the agriculturists; keeping in mind the findings of the present research study and previous related literature, the seed priming technique could open a new avenue to a sustainable agricultural system by increasing the production and improving the abiotic stress tolerance potential of economically important crops. In addition, the seed priming technique could further be extended to replace the conventional agricultural practices; however, investigating and ensuring its biosafety in living systems should be taken into consideration; therefore, there is a dire need for further studies to ensure its safe applicability for sustainable production of crops.

## Data Availability

All data generated or analyzed during this study are included in this published article.

## Notes

The seedlings of the maize were taken from the University of Peshawar, Pakistan, where the experiment was performed. All the experiments were performed in accordance with relevant guidelines and regulations.
